# Health professionals’ experience of treatment of patients whose community treatment order was revoked under new capacity-based mental health legislation in Norway: qualitative study

**DOI:** 10.1192/bjo.2022.592

**Published:** 2022-10-11

**Authors:** Nina Camilla Wergeland, Åshild Fause, Astrid Karine Weber, Anett Beatrix Osnes Fause, Henriette Riley

**Affiliations:** Division of Mental Health and Substance Abuse, University Hospital of North Norway, Tromsø, Norway; and Department of Health and Care Sciences, Faculty of Health Sciences, UiT The Arctic University of Norway, Tromsø, Norway; Department of Health and Care Sciences, Faculty of Health Sciences, UiT The Arctic University of Norway, Tromsø, Norway; Division of Mental Health and Substance Abuse, University Hospital of North Norway, Tromsø, Norway; Elden Law Firm, Tromsø, Norway

**Keywords:** Capacity-based legislation, capacity to consent, autonomy, coercion, community treatment order

## Abstract

**Background:**

Norway introduced capacity-based legislation in mental healthcare on 1 September 2017 with the aim of increasing patient autonomy and legal protection and reducing the use of coercion. The new legislation was expected to be particularly important for patients under community treatment orders (CTOs).

**Aims:**

To explore health professionals’ experiences of how capacity-based legislation affects healthcare services for patients whose compulsory treatment order was revoked as a result of being assessed as having capacity to consent.

**Method:**

Nine health professionals responsible for treatment and care of patients whose CTO was revoked owing to the new legislation were interviewed in depth from September 2019 to March 2020. We used a hermeneutic approach to the interviews and analysis of the transcripts.

**Results:**

The participants found that capacity-based legislation raised their awareness of their responsibility for patient autonomy and involvement in treatment and care. They also felt a need for more frequent assessments of patients’ condition and capacity to consent and more flexibility between levels of care.

**Conclusions:**

The study shows that health professionals found that capacity-based legislation raised their awareness of their responsibility for patient autonomy and involvement in treatment and care. They sought closer dialogue with patients, providing information and advice, and more frequently assessing patients’ condition to adjust treatment and care to enable them to retain their capacity to consent. This could be challenging and required competence, continuity and close collaboration between personnel in different healthcare services at primary and specialist level.

Capacity-based legislation has been introduced in several Western jurisdictions^1^ to enable healthcare for people with severe mental illness to comply with the Convention on the Rights of Persons with Disabilities.^2,3^ Norway introduced the legislation on 1 September 2017 to enhance patient autonomy and legal protection and reduce the use of coercion, particularly community treatment orders (CTOs).^4^

The Norwegian Mental Health Act now includes a requirement that a patient must clearly lack the capacity to consent, unless there is an imminent risk to the patient's life or the life and health of others (the harm criterion). ^5,6^ An assessment must be made of the patient's capacity to consent to voluntary admission and treatment. If the patient is considered to lack capacity to consent, involuntary admission and treatment must be implemented, regardless of whether the patient refuses or not.^5^ Four factors are emphasised in assessing capacity to consent: (a) the ability to understand information relevant to healthcare decisions, (b) the ability to apply the information to their own situation, especially in relation to their particular mental health problems and possible consequences of different treatment options, (c) the ability to use relevant information to weigh up treatment options and (d) the ability to express a choice.^7–9^ When patients have the capacity to consent, they have the right to refuse recommended treatment, but still have the right to receive the healthcare they need.^10^ Further, they are entitled to receive personalised information that provides greater insight into their condition and treatment options, which will enable them to be more involved in their own care and treatment.^10^

CTOs have been used in mental healthcare in Norway since 1961.^11^ They have been established following involuntary in-patient care when patients are considered to still need compulsory care and treatment, but as out-patients.^5,12^ A study from 2016 shows that CTOs were previously justified as ensuring maintenance treatment and preventing relapse,^12^ which is no longer possible when patients are considered capable to consent. In 2019, the prevalence rate of CTOs in Norway was 43/100 000 population.^13^

Before the new legislation, health professionals and family carers expressed concern that patients would refuse the treatment and care they needed and were worried about increased use of the harm criterion to justify CTO decisions.^4^ However, a study shows that 4 years after the change in the law, incidence rates and duration have not changed significantly, while prevalence rates have declined significantly and the use of the harm criterion has only shown a marginal increase.^13^

Organisation of CTOs and regulations on who may impose them vary between jurisdictions.^14^ The CTO regime in Norway is described in Rugkåsa et al^15^ and Wergeland et al.^16^ Norway has two levels of care: primary and specialist care. The person responsible for treatment, either a psychiatrist or a specialist clinical psychologist, makes CTO decisions under the Mental Health Act 1999.^5^ If this person considers medication to be necessary and the patient refuses, a separate decision is required for compulsory medication.^5^ Primary care staff are often responsible for implementation and daily care in connection with a CTO; this involves a general practitioner, mental healthcare, home care, staffed or unstaffed housing and various low-threshold services.^16^ Individuals with severe mental illness often need close monitoring to meet their basic needs and adjust treatment to their condition. The term condition indicates a temporary state of illness or health, and provides information about a patient's physical, mental and cognitive capacity at a specific point in time.^17^

The purpose of this study is to explore health professionals’ experiences of how capacity-based legislation affects healthcare provision for patients whose CTO was revoked after being assessed as capable of consent. The research question is: How do health professionals find that the new legislation affects treatment and care of patients whose CTO was revoked?

## Method

### Design

The study has a qualitative design, using in-depth interviews to explore health professionals’ experiences of the significance of capacity-based legislation for care of patients whose CTO has been revoked. The interviews and data analysis were inspired by a dialogical hermeneutic approach described by Fleming et al.^18^ This paper is part of a larger study which also explores patients’^16^ and family carers’ experiences.

### Study setting

This study took place in the sparsely populated northernmost region of Norway. Primary (municipal) healthcare includes general practitioners, home nursing and housing. The University Hospital of Northern Norway and Finnmark Hospital Trust provide specialist care in mental health and substance misuse in the region. The region has nine community mental health centres offering specialist care in an in-patient ward and an out-patient clinic. Outreach services are also available. Low population density and vast distances mean that some patients live several hours’ drive from the nearest mental health centre and have to fly to the nearest hospital.

### Patient and family carer involvement

As part of the larger study, four focus group interviews had been conducted with various groups affected by the change in legislation to gain insight into their expectations and opinions. These interviews were divided into distinct groups to explore participants’ opinions on what the change would mean for practice and what they thought the study should investigate. The participants in the four focus groups were divided as follows: Group 1 had personal experience of having been under a CTO and coercion, Group 2 consisted of relatives of former or current CTO patients, Group 3 consisted of specialist care staff with experience of CTO patients, and Group 4 contained primary care staff with experience of CTO patients. The focus group interviews were analysed with the aim of formulating the research questions and preparing interview guides.

At the start of the larger study, a peer group of six persons was also established; some members had been CTO patients, while others had experience as family carers of CTO patients. The peer group made suggestions for the research questions, interview guides and data collection. Owing to the COVID-19 pandemic, this group was not included in the analysis as originally planned.

### Recruitment

Participants in the present study were therapists or staff involved in the daily care of patients who had come off a CTO, having been assessed as capable of consent. Recruitment was conducted in a substudy that dealt with patients’ experiences of the new legislation;^16^ patients were asked whether one of their therapists or care workers could be invited to participate in the study. Ten out of twelve patients agreed to this. Following the patient's consent, the first author (N.C.W.) phoned the person to provide study information and invite the person to participate. All agreed to participate, and no participants later withdrew. COVID-19 prevented the interview of one participant who had agreed to be interviewed.

### Participants

Nine health professionals were interviewed in the study – seven women and two men – and the age range was from about 30 to 60 years. Four worked on a daily basis with mental health and substance misuse patients in primary care, either in home care or in sheltered housing. Five were therapists in in-patient or out-patient specialist healthcare. They were qualified as psychiatrists, social workers, healthcare assistants, environmental therapists and nurses with various specialisations. Most had extensive experience of working with people with severe mental illness under a CTO.

### Interviews

The first author (N.C.W.) conducted the interviews at the participants’ workplaces between September 2019 and March 2020. The 50–90 min interviews were audiotaped and later transcribed and anonymised. After each interview, the interviewer made notes about the interview situation and her perception of the interview.

The interview guide contained three main parts, with different subquestions and keywords. The first part was introductory questions concerning the presentation of the participant and the connection to the patient that was the inclusion criterion for participation. The main part contained questions about the participants’ experience of the change in the legislation and its impact on patient treatment, particularly regarding the patient who gave permission for their participation. The last part contained rounding off questions, including how the participants felt about the interview.

### Analysis

The empirical data were developed in dialogue between the participants’ descriptions of their experiences and the researchers’ understandings. A hermeneutically inspired process with repeated movement between the whole and parts was used to analyse the data and enhance understanding.^18^ The first author (N.C.W.) became well acquainted with the data by conducting, transcribing and anonymising all the interviews. The interviews were listened to and read based on the research question. Notes on a holistic understanding were written. Each interview was then read with a focus on concepts, sentences and passages, and on viewing these in light of the holistic understanding. Parts that answered or illuminated the research question were marked. We could then challenge and correct the first holistic understandings of the interviews to gain new understanding. Keywords for how the descriptions were understood and ideas, associations and possible themes were noted in the margin. This was repeated several times and the software program NVivo was used to organise the data. The meaning units were coded in NVivo, using the participants’ words and phrases as far as possible.^19,20^ NVivo mind maps were used for the visualisation of codes and categories.

In a hermeneutically inspired approach, researchers discuss their understandings of the findings and are open to different understandings of participants’ statements, which they try out in order to capture possible misunderstandings.^18^ Our extensive experience of similar work to that of the participants influenced how we as researchers were involved in interviews, transcriptions, analysis and presentation, and formed a sound basis for our understanding,^18,21^ since we have experience from clinical work, counselling, advocacy and legal assistance for patients in involuntary mental health treatment and CTOs.

Preliminary findings were sorted and categorised, and then discussed and interpreted by the research team in several rounds. Themes and concepts were tested to determine whether they could be understood differently and whether they were appropriate to the statements or categories, thus challenging our preunderstandings. The first author (N.C.W.) read the interviews several times to ensure that important statements and nuances were not overlooked. Quotes that best represented the themes were selected.

### Ethics

The authors assert that all procedures contributing to this work comply with the ethical standards of the relevant national and institutional committees on human experimentation and with the Helsinki Declaration of 1975, as revised in 2008. All procedures involving human subjects were assessed by the Regional Ethics Committee (REK Nord), REK No. 2018/1659, and approved by the data protection officer of the University Hospital of North Norway.

All participants received oral and written information about the study. They also received information on voluntary participation and the opportunity to withdraw from the study at any time before the data were included in the analysis, without giving any reason. The participants gave written informed consent to take part in the study. The participants’ names and sometimes also their gender have been changed for confidentiality.

The design and recruitment of the study meant that the participants were encouraged to talk about the patient who agreed to their participation. This necessitated a particularly respectful description of the patient.

### Presentation of results

The results consist of three main themes: (a) increased awareness of one's responsibility, (b) more frequent assessments of condition and capacity to consent and (c) the need for flexibility and continuity.

In the presentation of the results, participants are divided into two groups of health personnel based on their different duties, overall treatment responsibility or daily care provision.

## Results

### Increased awareness of one's responsibility

The participants providing daily care were positively surprised that most patients who had come off a CTO did not refuse the necessary healthcare, including medication. With some patients, there was a transition period where collaboration was challenging; these patients made choices that the health personnel disagreed with but had to accept. Anna, who had a patient who had been in involuntary treatment for several decades, put it this way:

‘We were all very worried! But things actually went very well. And it's still going well. We're very pleasantly surprised. I remember we were very … I thought this won't work, now he'll get ill, now he won't take his medicine. That was our main thought, that he wouldn't take the medication and how could he live in the housing then.’

The participants felt that it was right that patients with severe mental disorders should decide as much as possible about their lives and their treatment. Ina, a therapist, said:

‘I think it's important to realise that however ill people are, they're masters of their own lives. You shouldn't just come along [as a health professional] and tell them what they need and decide everything for them. It's important for them to decide for themselves as far as possible.’

A few patients refused all healthcare because they perceived the revocation of the CTO as meaning that they no longer needed medication or further care. Two of these patients had a severe relapse and were unable to receive help, which led to a new CTO for them.

All participants found it difficult to collaborate with patients whose severe mental illness sometimes made their symptoms increase and their capacity to consent decrease. Several participants stated that to improve collaboration, patients needed to feel that the treatment was useful and meaningful. Gry, a therapist, mentioned a patient who wanted help, but when she asked for it, she felt that health personnel misunderstood her or did not listen to her. The patient lost confidence in the healthcare services because she did not receive what she asked for, but had to accept treatment she did not agree with. Gry summed up the story as follows:

‘We have to be useful to people, offer them something meaningful, something they need.’

Detailed documentation requirements introduced with the new legislation were found to raise awareness of what decisions health personnel can make without strong justification. Tim, a therapist, said:

‘ … If you read old medical records, let's say the last 10 to 20 years, then I think, as an oversimplification, it might say: “The patient is ill. In my opinion, he needs medication. A CTO is needed”. But today we have to present the pros and cons (whether or not the patient has capacity to consent), what the patient wants, side-effects of medication and so on. The documentation requirements today obviously emphasise autonomy more. We don't treat them in such a patronising way now.’

Although several participants found that the documentation required much more of them, two pointed out the problem that patients receive the same written information on the decision. The decisions are difficult for patients to understand because the documentation requirements mean that the text is quite extensive and couched in legal and medical terminology.

Tim, a therapist, felt that managing involuntary treatment was a difficult task for society; following capacity-based legislation, a change in attitude was needed:

‘It's important to accept the change and not stick to a “take care of” attitude towards patients.’

Tim added that the shift from deciding what is best for patients to collaborating with them could be challenging for experienced professionals. He thought that the rules could be bent in line with therapists’ beliefs and attitudes, which would then colour their assessments.

### More frequent assessments of condition and capacity to consent

The participants providing daily care described how they assessed patients’ condition and helped them to make constructive choices about their treatment in order to maintain their capacity to consent. Anna systematically adapted daily care to facilitate collaboration. Her patient had lived in municipal housing for several years and had various physical conditions in addition to mental illness. Anna said that this meant that staff sometimes needed to be determined and help the patient to make decisions, regarding for example diet, personal hygiene and social skills. She said that the care she provided now was similar to the care she provided during the CTO, because they had known each other for many years:

‘ … he knows me very well. I may be a bit strict, I mean, I look after him properly, but I have such a good relationship with him. I make sure he's ok, like he gets the help he needs and I try to get my colleagues to give him the same care and … ’

The staff focused on providing personalised, repeated information to patients about their health, their rights and care and treatment options, to help them make sound choices to improve their health and maintain their capacity to consent.

Several participants found it difficult to determine whether patients understood the difference between compulsory and voluntary treatment. Siri, who provided daily care, mentioned a case where she became unsure of the patient's feelings about the situation:

‘She really wants to come off the medicine. But if she cuts it out too fast, she gets in such a state that she doesn't know how to live. And we got to a point where I had to intervene and say … her choice was between ending up on a CTO again and deciding to take the medicine after all. That was a critical point and I had to say, look, you've got to change your mind, or things won't be looking good for you! I didn't force her, but I spoke firmly … and I was a bit unsure about how much I could insist without forcing her. But that conversation boosted her trust again, and she listened to my advice. In her case, strong persuasion was needed and it wasn't about me or us wanting to force her to take the medication, but to help her to carry on. Take a bit more medicine now, so you can keep your freedom and your desire to come off it one day.’

Gry, a therapist, said the following about finding a balance between forcing patients and letting them decide for themselves:

‘It's a delicate balancing act. Especially with clients with bipolar disorder, where it can fluctuate a lot and if we discharge them too soon, they can mess things up for themselves, because I've seen several examples of that, which is very sad. Where they didn't get the care they needed and had to bear all the consequences themselves. It's important to see this from different angles. Even though we should have all respect for this [use of coercion], what we actually inflict on people.’

Gry found it demanding to be in situations where patients did not receive the necessary healthcare because they refused it.

All the therapists found it challenging to assess capacity to consent. The time frame and the assessment situation itself could jeopardise a thorough assessment, especially when the patient's condition could change rapidly. John said:

‘It's incredibly difficult! I have to try to find out what patients understand about their situation and their illness, and about what it means to consent … capacity to consent doesn't mean that you choose the same treatment as I recommend. … I think it's absolutely awful to have to write a good assessment in a short text, because it's really completely impossible. I think we often use our gut feeling about what's best, but we present all the arguments and write them in our assessments, but we can't really make brilliant assessments in such a short time.’

John described assessments as even more challenging when patients were taking drugs:

‘The ones who take drugs can often go in and out of capacity to consent and psychosis, and then you really have to change that text. You can't keep assessing every hour, that doesn't work.’

Birgitte, a therapist, was often dependent on clarifying a patient's condition with others who knew the patient well. However, this was not always possible in the limited time available. She explained:

‘You get a snapshot as a doctor. The patient may seem fine and doesn't need to be admitted to hospital. Then later home care gives you a completely different picture. Some of my patients may pull themselves together when they go to the doctor and they look very nice and proper. But if you're with them for more than 10 or 15 min, you see the delusions starting. These snapshots and capacity to consent don't match up. They should get information from people who know the patient, so that they can assess capacity to consent.’

### The need for flexibility and continuity

Both groups of participants pointed out the need for close collaboration between levels of care for patients whose CTO had been revoked. They found that collaboration was satisfactory for some patients. For others, resources were inadequate and they received poor-quality treatment and care. Participants from both groups wanted to be more accessible to patients. They called for more flexible use of health personnel in order to adapt treatment to patients’ condition and maintain their capacity to consent. Several mentioned the assertive community treatment (ACT) team, which has members from both primary and specialist care, and provides flexible care that the participants thought was suitable for the target group. Birgitte, a therapist, explained:

‘Most patients are offered care and treatment, and we [in specialist healthcare] can provide this, but they refuse it. In a busy day, it's easy to feel rejected. But this rejection is linked to paranoia and isolation. But you can also do what the ACT team does, they do a fantastic job. They keep on knocking at the door, maybe eighteen times until they see the curtains move. And the patient gets to know the voice and those are the kind of resources I think … Flexibility and the way they work … that's what I miss so much … I think we could ensure care quality and improve our patients’ quality of life.’

The participants were concerned that the vast distances in the region made it difficult to assess and treat patients whose CTO had been terminated. Several of the therapists found that the long distances limited their ability to take an active part in daily care and treatment, and that it was challenging to achieve good collaboration with patients who lived far from the hospital. The distances made it difficult to know whether treatment and care were being followed up in a satisfactory manner, and to know when the CTO should be continued or revoked. Gry felt that the therapeutic relationship was a vital factor in any decision to revoke a CTO:

‘I think it [a CTO] has been necessary in one phase at least. But I may well have been a bit too afraid to revoke it too soon, I mean, it may have been … perhaps looking back at it, I might have dared to cancel it sooner. But experience is also important here … assuming you've had good collaboration and a good relationship and so on, where both sides could clearly see that the CTO was no longer necessary.’

## Discussion

The aim of this study is to explore health professionals’ experiences of how capacity-based legislation affects healthcare services for patients whose CTO was revoked after being assessed as capable of consent. The results are discussed in light of the aim of the legislation to strengthen patient autonomy and legal protection, and reduce the use of coercion.

The study shows that health professionals have become more aware of how to ensure patients’ right to autonomy and involvement in their treatment. This is in line with government expectations and the aim of the legislation.^4^ When patients have the capacity to consent, health professionals see that a patient may choose treatment that differs from what is recommended, which they have to respect. They described a more equal dialogue with patients about treatment and care, which is in line with patients’ own experience.^16^ The participants were very keen, on a professional and personal level, for patients to manage without compulsory treatment, and saw the need for new forms of collaboration to make this possible.

Both groups of health personnel made efforts to achieve close communication with patients. To facilitate participation, they placed greater emphasis on providing patients with personalised information about their condition and treatment options. The more equal relationship resulted in more discussion and negotiation, which meant that the health professionals listened to what patients considered useful. They tried to respond to their wishes by presenting the advantages and disadvantages of different treatment options, while also making recommendations. Patient participation in dialogues about their treatment and care presupposes personalised information, which is mandated by law.^10,22^ Shared decision-making is emphasised by the Norwegian Directorate of Health as an important way of helping patients to make informed choices.^23^ However, one study finds that shared decision-making can be difficult to apply in practice; it is time-consuming and health professionals are unsure as to whether patients with psychotic disorders can understand information sufficiently well to make informed choices.^24^

Health professionals often find it difficult to balance care and control when treating patients under CTOs.^12^ If a patient has come off a CTO but still has a serious mental illness, health professionals try to find flexible ways to help the patient receive the same treatment and care without being too strict or controlling. They try to help patients to retain their autonomy through ‘compassionate interference’.^25^ Active and committed health professionals who would not leave patients to make their choices alone do not need to threaten autonomy with their interference. They might in fact be helping patients to retain or achieve autonomy. If patients have a firm conviction about their illness or their environment that is completely different from the therapist's understanding, communication and interaction can be challenging.^26^ The requirement in capacity-based legislation for increased patient autonomy represents an even greater challenge to health professionals when the patient's capacity to consent fluctuates in line with the illness.

This study shows that more frequent assessments of patients’ condition and capacity to consent are needed. Health personnel who provide daily care must handle complex and demanding care work over time. They described how care and treatment were adjusted according to the patient's condition. Because many patients are unable to ask for help when their condition worsens, care workers must monitor their condition and make daily assessments.^27^ Close monitoring and continuity are necessary to detect deterioration and intervene before the patient becomes so ill that coercion is needed. This requires close cooperation between health professionals. Interventions often involve negotiations with the patient and require a good relationship, which can be problematic when the patient has experienced coercion.^28^

Therapists responsible for assessing patients’ capacity to consent expressed concern about whether the assessments were thorough enough. They found that the assessment situation was often complicated by time pressure, fluctuations in the patient's condition, drug or alcohol addiction and poor knowledge of the patient coupled with lack of contact with someone more familiar with the patient. Previous studies show that therapists have attached great importance to CTOs to improve patients’ health, and have therefore maintained the CTO in order to ensure stability and avoid relapse.^12^ Capacity-based legislation requires therapists to recognise the patient's right to self-determination and facilitate a more equal dialogue. Since the Mental Health Act 1999 has now established the right of patients to decide on their treatment and daily life,^5^ the quality of the assessment of capacity to consent is of vital importance for the patient's legal protection.^29^ The assessment is discretionary^30^ despite the availability of assessment tools.^7,8^

Patients have a legal right to receive necessary healthcare at both primary and specialist levels.^10^ Studies conducted before capacity-based legislation was introduced show that the range of services decreased at both levels when a CTO was revoked.^31,32^ The finding in the present study that the daily care provided today is similar to that previously provided to the same patient under a CTO may suggest that the new legislation has led to a change in clinical practice. Based on their experiences following the legislation, both groups of health personnel called for more flexibility in the organisation of staff resources in order to adapt treatment to patient needs in ways that promote autonomy. This is in line with studies that show that lack of resources and flexibility in healthcare can increase the risk of involuntary hospital admission^33^ and that there is a need for easy access to healthcare in the early stages of deterioration.^34^ In the present study, both groups underlined the importance of maintaining significant relationships and called for frameworks that allow for continued contact with patients even when they need treatment and care from other health service providers for shorter or longer periods.

The study shows that the participants considered it important to be able to offer healthcare on the patient's terms with more flexible working methods across levels of care. However, this presupposes a safe and stable working environment to enable health personnel to maintain their commitment and cope with challenging situations.

### Strengths and limitations

The interviews were conducted 24–30 months after capacity-based legislation was introduced. The participants had therefore gained experience of the new scheme, but had not had sufficient time to establish it as a well-tried practice. The interviews were conducted at a time when the change was the subject of much reflection and discussion in both groups of health personnel. This probably enriched the descriptions of experience for the study.

The study had a small number of participants, but they provided different healthcare services and were from urban and rural areas, which gave a variety of descriptions of experience, but from a single region. It is a weakness that no general practitioners participated in the study because they are part of the care team for all patients, and make assessments of capacity to consent.

## Data Availability

To protect the anonymity of the participants, the data on which this paper is based will not be made generally available, with the exception of the data that have been carefully selected for presentation in the paper.
